# Non-invasive Ventilation and High-Flow Nasal Cannula in Head/Brain Injury with Risk of Pneumocephalus: Is There a Potential Application?

**DOI:** 10.5152/TJAR.2023.21116

**Published:** 2023-04-01

**Authors:** Güniz M. Köksal, Çiğdem Akyol Beyoğlu, Mohamad El-Khatib, Manuel Á. Gómez-Ríos, Peter Papadakos, Antonio M. Esquinas

**Affiliations:** 1 Department of Anaesthesiology and Reanimation, İstanbul Cerrahpasa University, Cerrahpasa Medical Faculty, İstanbul, Turkey; 2 Department of Anaesthesiology, American University of Beirut-Medical Center, Beirut - Lebanon; 3 Department of Anaesthesiology and Perioperative Medicine, Complejo Hospitalario Universitario de A Coruña, A Coruña, Galicia, Spain; 4 Intensive Care Unit, Hospital M. Meseguer, Murcia, Spain; 5 Intensive Care Unit and Non Invasive Ventilatory Unit, Hospital M. Meseguer, Murcia, Spain

**Keywords:** Brain surgery, complications, head injury, NIV, pneumocephalus, trauma

## Abstract

Non-invasive ventilation application in neurocritical care with risk of pneumocephalus is controversial. Non-invasive ventilation-related increased intrathoracic pressure increases intracranial pressure via direct transmission of intrathoracic pressure to the intracranial cavity. In addition, increased thoracic pressure decreases venous return to the heart and increases vena jugularis interna pressure, thereby increasing cerebral blood volume.

Pneumocephalus is one of the major concerns after non-invasive ventilation application in head/brain trauma patients. Non-invasive mechanical ventilation may be performed in limited conditions in head trauma/brain surgery with appropriate and close monitoring. High-flow nasal cannula oxygen therapy can provide higher FiO_2_ as manifested by a larger increase in PaO_2_/FiO_2_ ratio and provide the theoretical basis in pneumocephalus because augmenting the PaO_2_ more effectively would accelerate nitrogen (N_2_) washout.

As a result, non-invasive mechanical ventilation may be performed in limited manner in head trauma/ brain surgery with appropriate and close monitoring.

**Main Points**

- Non-invasive mechanical ventilation may be used in head trauma/brain surgery with dural injury in limited conditions with appropriate and close monitoring.
- Patients under treatment with continue positive airway pressure and bi-level positive airway pressure may develop pneumocephalus.
- High-flow nasal oxygen therapy may be a choice in head trauma/brain surgery patients.

## Introduction

Mechanical ventilation is an artificial positive pressure support for respiration to assist breathing in case of respiratory failure (either hypercapnic or hypoxemic). It reduces the patient’s work of breathing by overcoming excessive imposed loads (e.g., abnormal airways resistance and/or lung compliance) with subsequent improvements of oxygenation and carbon dioxide elimination.^1^ Non-invasive ventilation (NIV) may provide optimum respiratory support to patients thus avoiding the complications of invasive mechanical ventilation including barotrauma (pneumothorax), volutrauma, biotrauma, ventilator-associated pneumonia and sinusitis, delirium, decreased cardiac output, fluid retention, pulmonary emboli and deep venous thrombosis.^2,3^ Recently, NIV has been suggested as a treatment option in patients with trauma, acute respiratory insufficiency and in the early stage of “Acute Respiratory Distress Syndrome” (ARDS).^4^ However, the NIV application in these conditions is still a matter of discussion in the scientific literature.^5^

After an initial cranial defect, air can move into the brain through a paranasal sinüs or the mastoid cells, which can be aggravated by swallowing, coughing or sneezing under a pressure greater than that within the cranium.^6^ Air moves into the cranium *via* a fistula which acts as a 1-way valve. A decreased cerebrospinal fluid-related decreased intracranial pressure supports air bubbles wiggling through the cranium. Also, increased thoracic pressure decreases venous return to the heart and increases vena jugularis interna pressure, thereby increasing cerebral blood volume.^7^

It is important to consider the mechanism and extension of injury when deciding to administer NIV in trauma patients. For example, head, neck and middle face traumas are unsuitable for administration of NIV. Furthermore, high consciousness levels and integrity of the upper airways should be ensured in such cases.^8^ The main goal in managing head trauma/brain surgery patients must be to protect the patient from secondary damage, primarily cerebral ischaemia of the primary injury. Secondary damage occurs due to hypoxia, hypercapnia, hypotension, hypoglycaemia and hyperpyrexia, especially in head/brain trauma patients. Therefore, management of head trauma/brain surgery patients is targeted at optimising cerebral perfusion, oxygenation, and avoiding hypoxia and hypercapnia.^9^

The aim of this narrative review is to emphasise the aspects of NIV treatment which may not be reviewed in the literature in this controversial use of NIV in head injury. 

### Clinical and Research Consequences

#### The Rationality of Non-Invasive Ventilation Use in Head Trauma/Brain Surgery and Controversy of its Safety

In patients with head trauma/brain surgery who are conscious in the perioperative period, NIV can be applied to prevent hypoxia (maintain an arterial PaO_2_ above 11 kPa) and hypercapnia (maintain an arterial PaCO_2_ between 4.5 and 5 kPa) and to avoid intubation.^10,11^ Before considering NIV on such patients, it is necessary to determine whether the patient’s upper airway is compromised or not. It must be kept in mind that NIV [continue positive airway pressure (CPAP) and bi-level positive airway pressure (BIPAP)] application to prevent respiratory failure may cause pneumocephalus (PNC) which indicates the presence of air in the intracranial cavity.^12^

Kopelovich et al^13^ applied CPAP/BIPAP in the post-operative period for a patient with obstructive sleep apnea (OSA) who underwent trans-sphenoidal resection. At the end of 2 weeks, PNC developed in the patient. Chee BNH et al^14^ performed CPAP in the post-operative period because of OSA in patients with ventriculo-peritoneal shunting after trauma and surgery. Continue positive airway pressure application was discontinued due to developing rhinorrhoea in these patients, and the defect was surgically closed afterwards.

If there is a pathology in the area close to the air inlet during NIV application or if the operation is performed in these areas (such as paranasal sinus, skull base, pituitary fossa, middle ear, and soft tissue defects in the head/neck), post-operative NIV applications should be carefully monitored. Non-invasive ventilation applications can be performed more safely after 6 weeks of dural repair.^14^

In 2008, Huncke et al^15^ reported their findings on the use of CPAP in a morbidly obese patient with OSA who underwent an awake craniotomy. They stated that the patient who was under sedation with dexmedetomidine remained comfortable and cooperative during the operation. Lee et al^16^ applied CPAP during the perioperative period with a mask under deep sedation to patients with severe chronic obstructive pulmonary disease (COPD) undergoing frontal craniotomy. This procedure is called “awake operation” which does not require a deep sedation. They reported that this application was very effective and that intraoperative NIV administration prevented the development of hypoxemia and hypercarbia. They did not observe any post-operative complications. In another study, patients with OSA under CPAP treatment were divided into 2 groups after trans-sphenoidal pituitary resection.^17^ One group received CPAP in the post-operative period, while the other group did not, and there were no differences between the 2 groups in terms of post-operative complications.

In the British Thoracic Society guidelines, NIV application in multi-trauma patients with hypoxemic respiratory failure is recommended and rated as C-level and had low-grade evidence.^18^ The Canadian Critical Care Trials Group and the Canadian Critical Care Society Non-invasive Ventilation guidelines do not recommend NIV application in similar cases due to insufficient findings and controversies.^19^

A pressure of around 15 cmH2O applied to the upper airways by a NIV treatment which is close to the intracranial pressure makes no difference. In the absence of a previous injury, this level of pressure is unlikely to have any effect on air intake into the CNS. However, air can enter the intracranial compartments directly through the fractures of the cranial bones and sinuses as a result of trauma and the defect created by a penetrating body. In such a case, the positive pressure effect of NIV will facilitate air entry through the existing defect. It has been shown that positive pressure applied to the upper airways, even for a short time, can cause pneumocephalus in patients with previous sphenoid sinus bone rupture.^13^

Continue positive airway pressure can increase intrathoracic pressure resulting in a decrease in venous return and cardiac output. A decreased venous return may result in an increase in intracranial pressure and together with a decreased cardiac output following an undesirable negative effect on cerebral blood flow that may arise. 

Non-invasive ventilation treatment in patients with head trauma/brain surgery who require mechanical respiratory support should exclude patients with a reported or suspected fractures of the skull of cranial bones and sinuses, dural defects or fistulas. 

#### Current Evidence in the Use of Nasal High-Flow Oxygen Therapy in Head Trauma/Brain Surgery

When compensatory mechanisms such as hypoxic pulmonary vasoconstriction become insufficient, pulmonary and extra-pulmonary damage can potentially lead to increased morbidity and mortality.^20^ The administration of nasal high-flow oxygen therapy, another form of non-invasive ventilatory support, may help respiratory mechanics by improving ventilation and increasing oxygenation. It provides nasal respiratory support with optimally heated and humidified oxygen at high-flow rates (up to 60 L per minute).^21^ In high-flow applications, tidal volume is increased and end-tidal expiratory lung volumes have been shown to improve oxygenation. Moreover, it decreases respiratory rate proportionally to the delivered flow rate.^22^ High-flow nasal cannula (HFNC) oxygen therapy can provide higher FiO_2_ as manifested by a larger increase in PaO_2_/FiO_2_ ratio and provide the theoretical basis for using HFNC oxygen therapy in PNC because augmenting the PaO_2_ more effectively would accelerate N_2_ washout. Based on a previous mathematical model of the rate of PNC absorption, HFNC oxygen therapy can theoretically outperform other conventional oxygen therapy devices such as non-rebreather mask (NRB).^23-26^ However, it has also been reported that it does not affect atelectasis compared to standard oxygenation. However, it prevents the closure of airways and prevents dead space ventilation. This delivers high mean airway pressure. Thus, the work of breathing during HFNC therapy is reduced.^27^ There may be improved intracranial compliance. Pneumocephalus has not been reported in any head trauma/brain surgery patient who has applied for HFNC oxygen therapy.

Wong et al^28^ applied high-flow intranasal oxygen to a patient with OSA who underwent awake craniotomy. Oxygen and carbon dioxide partial pressure levels remained normal under deep sedation. The patient and the surgeon were highly satisfied. No complications were observed in the post-operative period.

Additionally, HFNC oxygen therapy may be the ideal mode of oxygen delivery in cases of post-operative PNC. Siegel et al^29^ presented 3 cases of post-operative PNC who were symptomatic. All patients improved both clinically and radiographically within a few hours with the administration of HFNC oxygen therapy, faster than in both anecdotal experience and published trials due to its steady FiO_2_ administration, positive pressure, comfort and low side-effect profile. 

Though the concept of pulmonary oxygen toxicity has been well described,^30^ there are no reports of oxygen toxicity with the use of HFNC. Otherwise, there are no guidelines regarding contra-indications of HFNC oxygen therapy, but considerations should be taken as with other modes of positive pressure NIV (decreased consciousness, claustrophobia, airway obstruction, facial injury or malformation, copious sputum, high risk of aspiration, unstable haemodynamics and respiratory arrest).^31^

On the other hand, tension PNC with severe neurological sequelae was a rare complication in HFNC in paediatric population receiving respiratory support with HFNC oxygen therapy.^32-35^ Nevertheless, it needs to be considered, as early diagnosis and appropriate treatment can modify prognosis. There is no sufficient data about HFNC safety of use in head trauma/brain surgery and further research is needed to evaluate its safety and effectiveness and the frequency of serious adverse events. The resulting knowledge should be used to develop clear guidelines and protocols for the use of HFNC oxygen therapy in such situations.

As a result, NIV can be applied in conscious and cooperative patients without head and neck trauma, deformed bone and soft tissue damage in the face. In these patients, NIV administration is not recommended in the guidelines as reflected by a “low-grade recommendation” for the use of NIV in trauma patients by the British Thoracic Society guidelines and “no recommendation” by Canadian Critical Care Trials Group/Canadian Critical Care Society NIV Guidelines Group because there is still much disagreement on this issue. Patients undergoing NIV should be monitored strictly because PNC may develop. In recent years, nasal high-flow oxygen application is encouraged in head trauma/brain surgery patients with no complications observed in the post-operative period. Further prospective, randomised, clinical studies are needed on this topic.

Table 1 suggests a comparative use of both NIV and HFNC techniques during head trauma/brain surgery.

## Conclusion

Non-invasive mechanical ventilation may be performed in limited conditions in head trauma/brain surgery with dural injury with appropriate and close monitoring. During NIV application in patients with head trauma/brain surgery, a close serial neurologic examination based on consciousness and changes in exam should be done in addition to haemodynamic, and respiratory monitoring should be performed. There are many reported cases of PNC developing in patients with CPAP and BIPAP treatment. High-flow nasal oxygen therapy may be a choice in head trauma/brain surgery patients. Further prospective, clinical, randomised controlled trials are needed in this subject.

## Figures and Tables

**Table 1.** Comparison of NIV and HFNC Treatment During Head Injury/Brain Surgery

	NIV	HFNC
Advantage	Safely supports ventilation excluding hypoxia, hypercarbia Enables neurologic assessment	Embetters PNC No clear contraindication
Disadvantage	Close monitoring against risk of secondary neurologic damage Poor data available about safety in head/brain injury	Unable to treat atelectasis No data available about safety in head/brain injury

HFNC, high-flow nasal cannula; NIV, non-invasive ventilation; PNC, pneumocephalus.
